# Supplementary material for: Reconciling heterogeneous dengue virus infection risk estimates from different study designs
Source: Proc Natl Acad Sci U S A. 2024 Dec 31;122(1):e2411768121. doi: 10.1073/pnas.2411768121 (PMC11725863; doi:10.1073/pnas.2411768121)
Supplement: Supplementary file 1 — Appendix 01 (PDF) [file pnas.2411768121.sapp.pdf]

PNAS

Supporting Information for

Reconciling heterogeneous dengue virus infection risk estimates from different study designs

Angkana T. Huang, Darunee Buddhari, Surachai Kaewhiran, Sopon Iamsirithaworn, Direk Khampaen, Aaron Farmer, Stefan Fernandez, Stephen J. Thomas, Isabel Rodriguez Barraquer, Taweewun Hunsawong, Anon Srikiatkhachorn, Gabriel Ribeiro dos Santos, Megan O'Driscoll, Marco Hamins-Puertolas, Timothy Endy, Alan L. Rothman, Derek A. T. Cummings, Kathryn Anderson, Henrik Salje

Angkana T. Huang and Henrik Salje
Email: ah2223@cam.ac.uk; huangat@gmail.com; hs743@cam.ac.uk

This PDF file includes:

Figures S1 to S11
Tables S1 to S7
SI References
Supplementary Mathematical Analysis

Data descriptions

Table S1. Descriptions of serological data included in this study.

**Cohort study** (study population)	**Years**	**Ages**	**N**
**KPS1** (Primary school children)	1998 to 2002	4 to 16	35,213 blood samples<br>3,436 individuals
**KPS2** (Primary school children)	2004 to 2008	3 to 15	3,180 blood samples<br>16,201 individuals
**KPS3** (Community children)	2010	3 to 11	1,659 blood samples<br>16,201 individuals
**KFCS** (Mother-infant and their multigenerational family members)	2016 to 2019	3 to 30	3,491 blood samples<br>1,116 individuals<br>318 family clusters

Standard models in FOI inferences

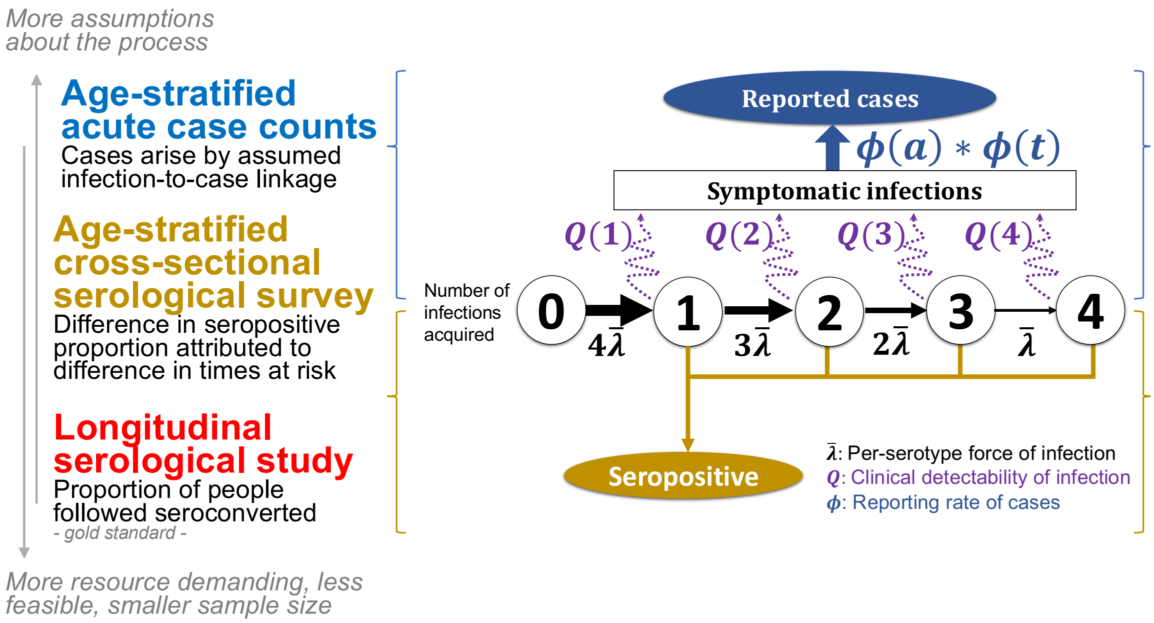

Figure S1. Diagram of standard models linking underlying infection process to observed data.

Table S2. Descriptions of parameters in the standard model linking force of infection to the number of dengue cases that sought care at Kamphaeng Phet Hospital (KPPH).

Parameter	Description	Prior	Prior justification
$\bar{\lambda}(t)$	Annual per-serotype force of infection	Exponential(2)	Weak prior
$p_{severe}(1)$	Probability that 1st infections of individuals resulted in severe infections relative to 2nd infections	Beta(1, 9)	Weakly informative prior to encode knowledge of 1st dengue infections being relatively mild compared to 2nd infections
$p_{severe}(i)$	Probability that i-th infections of individuals resulted in severe infections relative to 2nd infections	Beta(1, 19)	Informative prior to reflect the rare occurrence of severe dengue in 3rd and 4th infections
$\phi(a)$	Probability that a severe case of age a sought care at KPPH relative to cases of age 0-2yrs (reference class)	Lognormal(0, 0.1)	Informative prior to favor reporting to be constant in age unless data suggests otherwise
$\phi(t)$	Probability that a severe case at age 0-2yrs sought care at KPPH at time t	Beta(2,2)	Weak prior

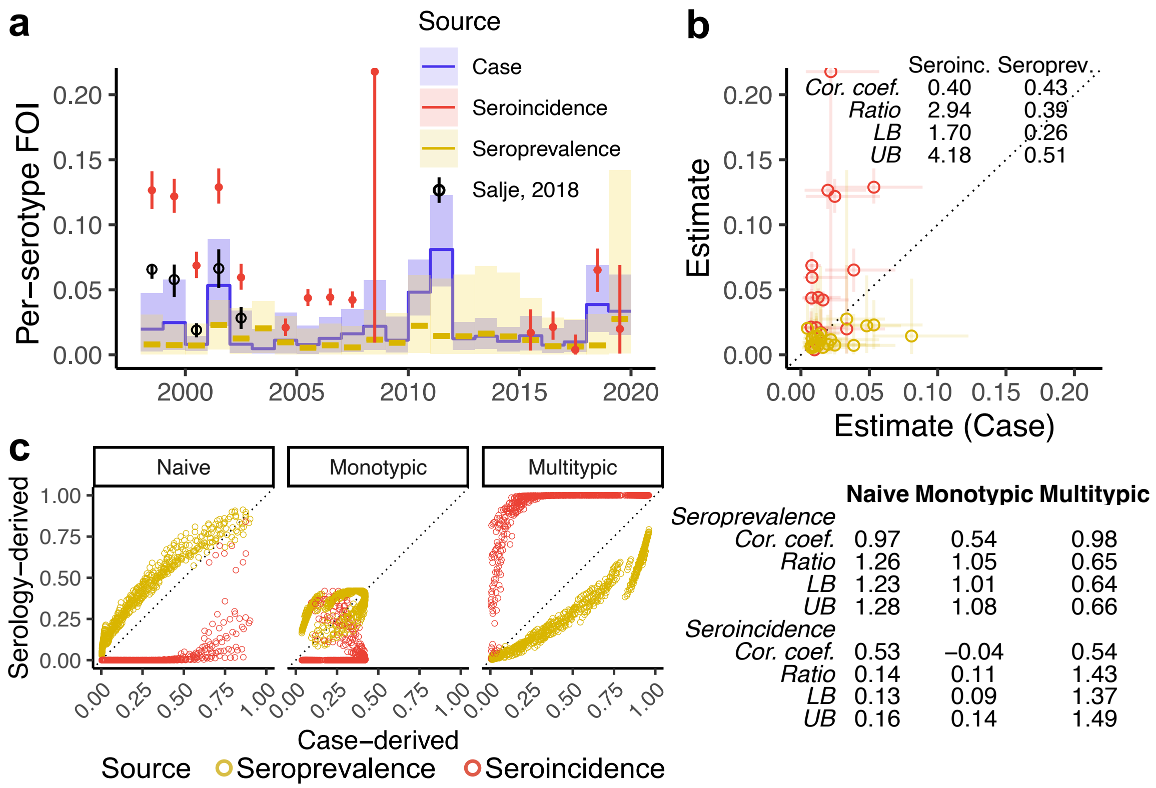

Figure S2. Estimates from standard force of infection (FOI) inference models when using seropositivity threshold of 20. a) Annual FOI estimated from each of the data sources: seroincidence data (red) and seroprevalence data (yellow) using seropositivity threshold of GMT>=20, and case data. **b)** Seroincidence-derived (red) and seroprevalence-derived FOI (yellow) compared against case-derived FOI (x-axis) and **c)** relationships between the respective susceptibility reconstructions. Each point in the reconstruction represents the proportion in each age-year that has not been infected with DENV (naive), has been infected by one serotype (monotypic) or more than one serotype (multitypic). LB=Lower bound, UB=Upper bound of the 95%CI of ratios.

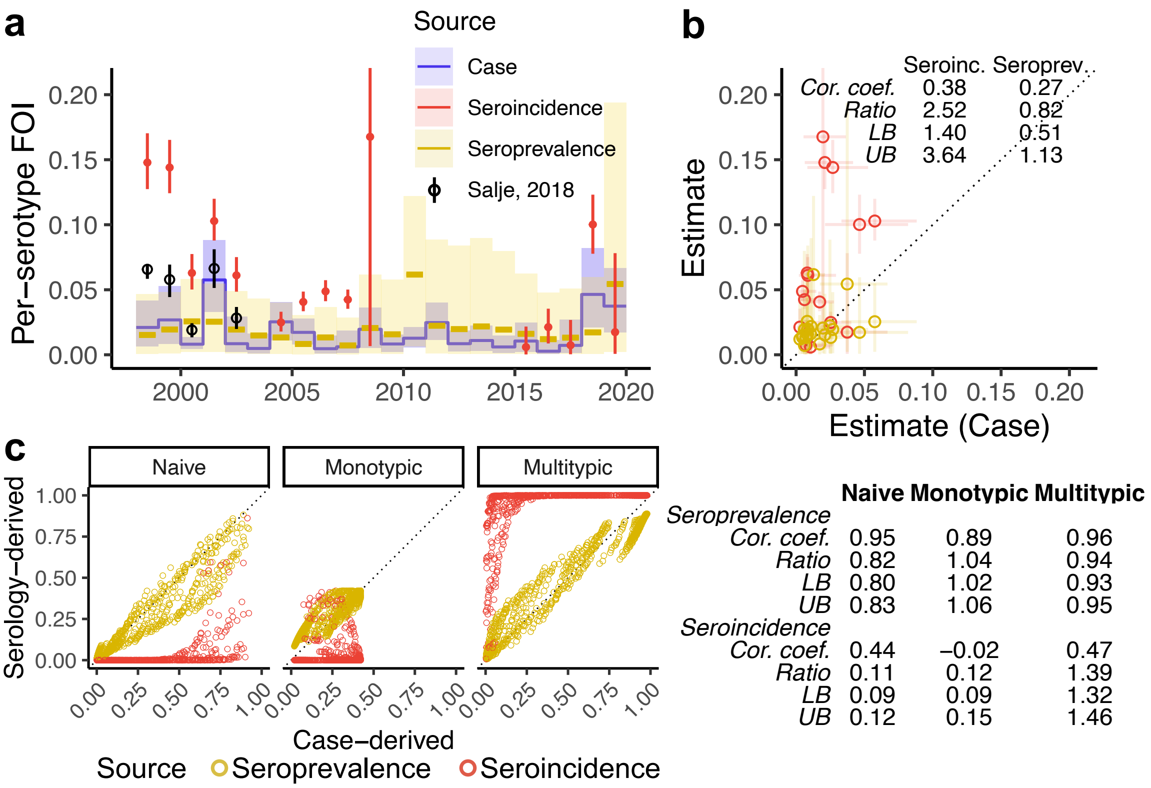

Figure S3. Case-derived force of infection (FOI) inferred from lab-confirmed cases compared against seroprevalence and seroincidence derived FOIs when using seropositivity threshold of 10. **a)** Annual FOI estimated from each of the data sources: seroincidence data (red) and seroprevalence data (yellow) using seropositivity threshold of GMT>=10, and lab-confirmed case data. **b)** Seroincidence-derived (red) and seroprevalence-derived FOI (yellow) compared against case-derived FOI (x-axis) and **c)** relationships between the respective susceptibility reconstructions. Each point in the reconstruction represents the proportion in each age-year that has not been infected with DENV (naive), has been infected by one serotype (monotypic) or more than one serotype (multitypic). LB=Lower bound, UB=Upper bound of the 95%CI of ratios.

Simulations to study effects of model assumption violations

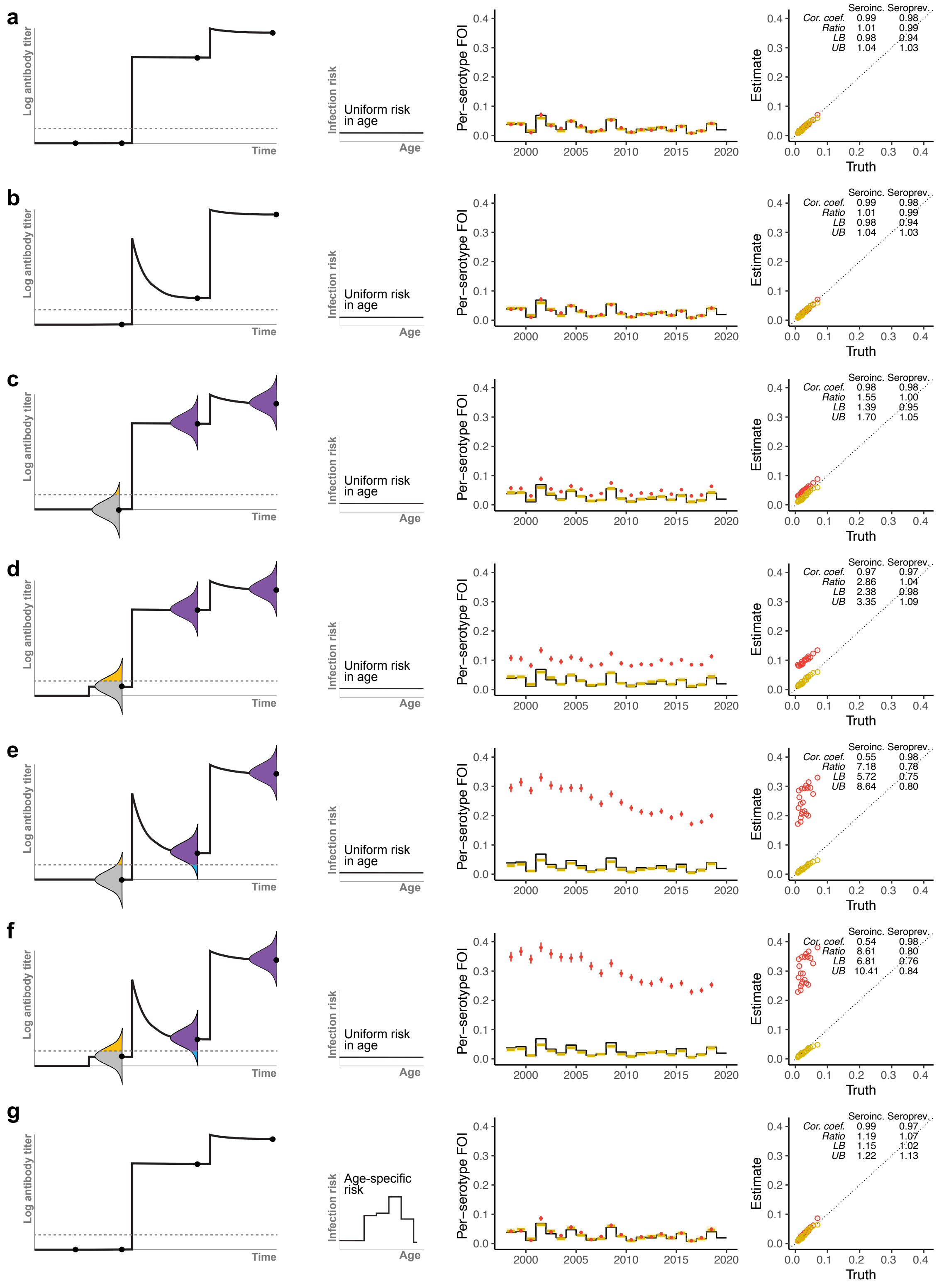

Figure S4. Effects of violating model assumptions on inferred infection risk in highly powered datasets using standard serological models at a low seropositivity threshold (GMT>=10). Left of each panel are schematics of assay variability, antibody kinetics, and

seropositivity thresholds used to simulate the data: **a)** Assay without noise, durable monotypic titers, without cross-reactive (CXR) titers, **b)** assay without noise, waning monotypic titers, without CXR titers, **c)** noisy assay, durable monotypic titers, without CXR titers, **d)** noisy assay, durable monotypic titers, with CXR titers, **e)** noisy assay, waning monotypic titers, without CXR titers, **f)** noisy assay, waning monotypic titers, with CXR titers. All of which infection risk is uniform in age. **g)** Assay without noise, durable monotypic titers, without CXR titers, but infection risk is non-uniform in age. Center of each panel compares inferred force of infection from seroincidence data (red) and seroprevalence data (yellow) to ground truth (black). Right of the panels are scatter plots between inferred infection risk and true infection risk by age.

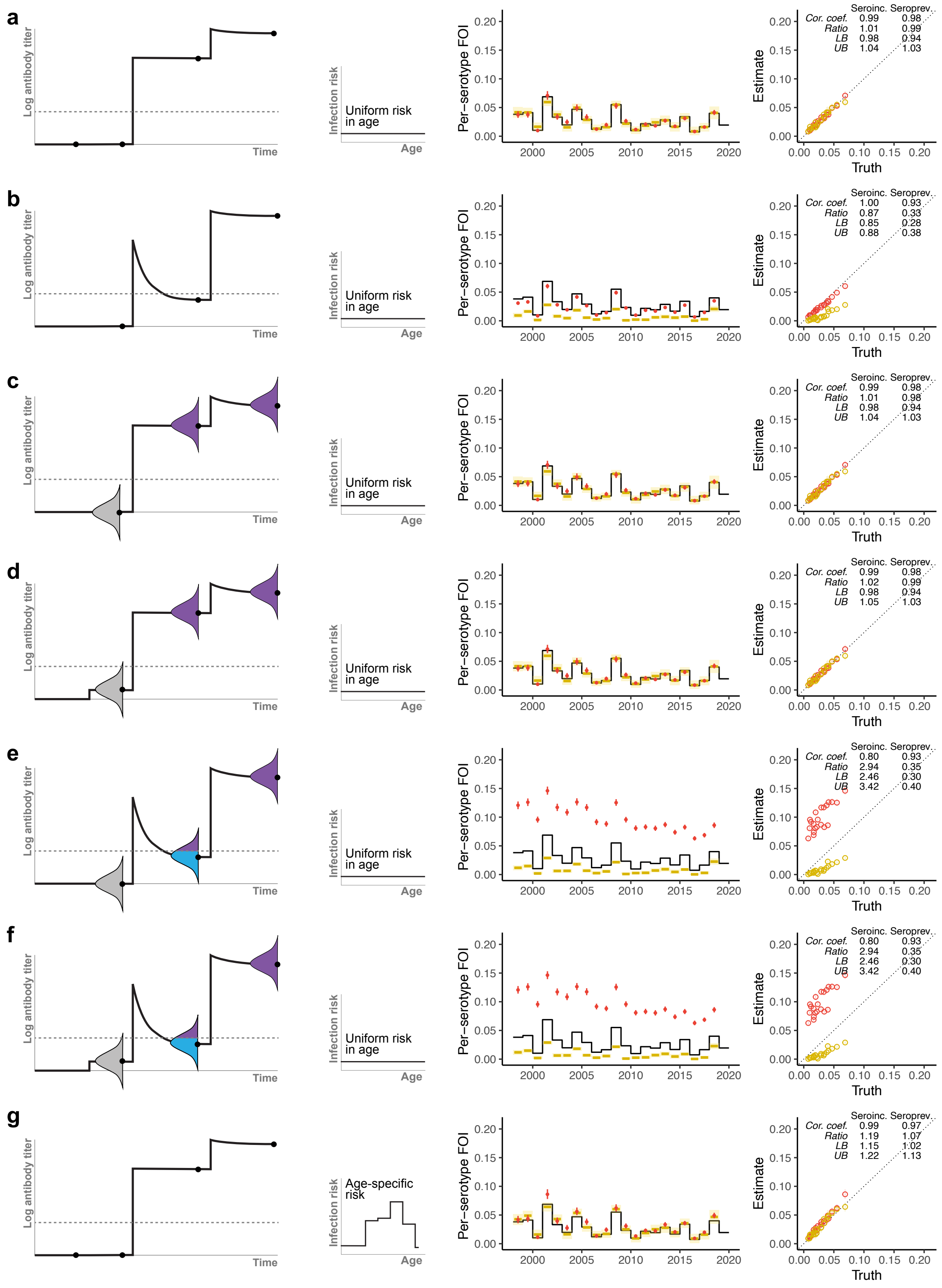

Figure S5. Effects of violating model assumptions on inferred infection risk in highly powered datasets using standard serological models at a high seropositivity threshold (GMT>=20). Left of each panel are schematics of assay variability, antibody kinetics, and

seropositivity thresholds used to simulate the data: **a)** Assay without noise, durable monotypic titers, without cross-reactive (CXR) titers, **b)** assay without noise, waning monotypic titers, without CXR titers, **c)** noisy assay, durable monotypic titers, without CXR titers, **d)** noisy assay, durable monotypic titers, with CXR titers, **e)** noisy assay, waning monotypic titers, without CXR titers, **f)** noisy assay, waning monotypic titers, with CXR titers. All of which infection risk is uniform in age. **g)** Assay without noise, durable monotypic titers, without CXR titers, but infection risk is non-uniform in age. Center of each panel compares inferred force of infection from seroincidence data (red) and seroprevalence data (yellow) to ground truth (black). Right of the panels are scatter plots between inferred infection risk and true infection risk by age.

Efficiency in correcting for model violations

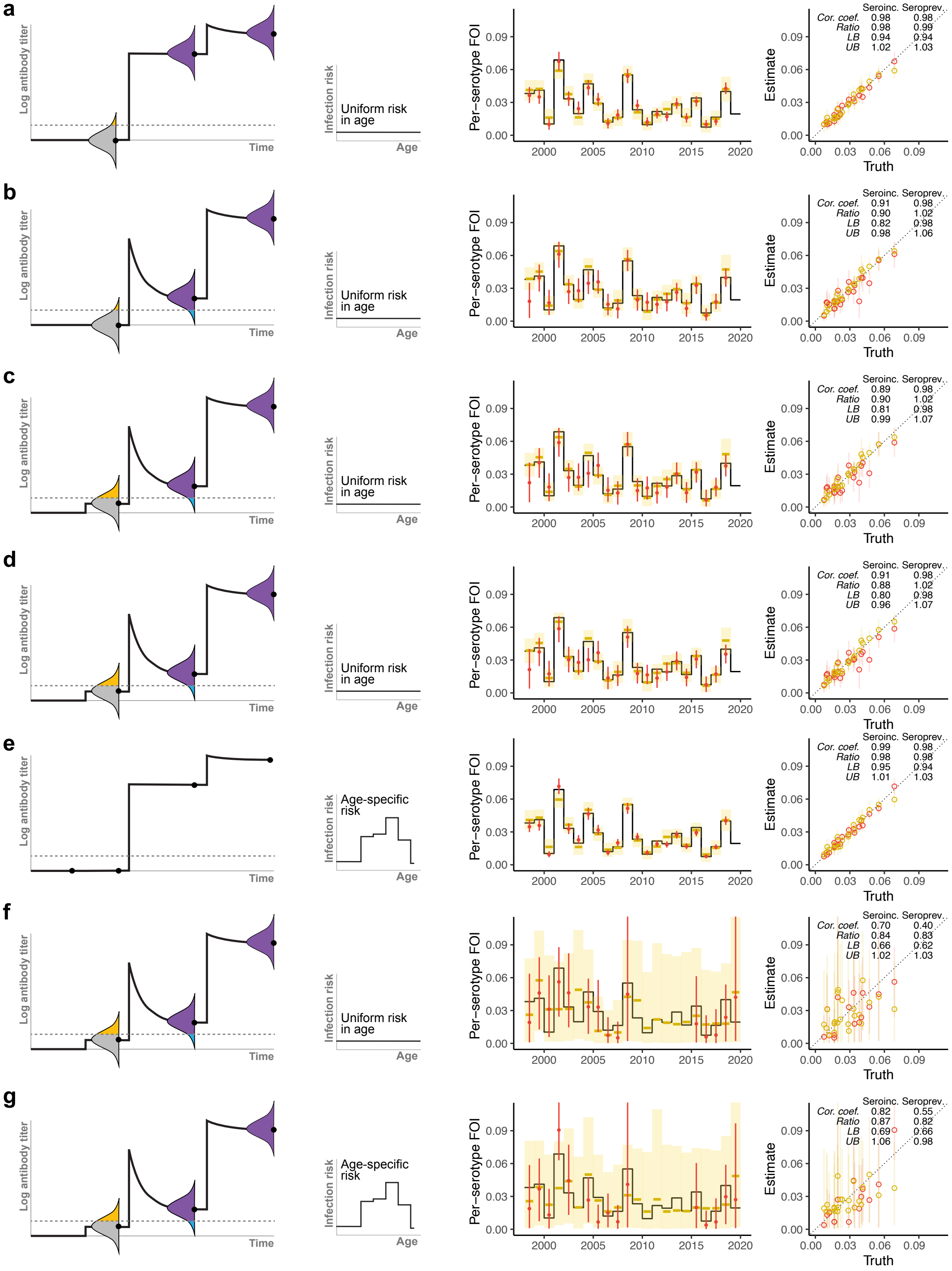

Figure S6. Efficiency in correcting for model assumption violations to recover true temporal infection risk when using a low seropositivity threshold (GMT>=10). Left of each panel are schematics of assay variability, antibody kinetics, seropositivity thresholds, and age-

specific infection risk used to simulate the data: **a)** noisy assay, durable monotypic titers, without cross-reactive titers, uniform risk in age **b)** noisy assay, waning monotypic titers, without cross-reactive titers, uniform risk in age **c)** noisy assay, waning monotypic titers, with cross-reactive titers, uniform risk in age **d)** noisy assay, waning monotypic titers, with cross-reactive titers, non-uniform risk in age, **e)** assay without noise, durable monotypic titers, without cross-reactive titers, non-uniform risk in age. All of which were simulated as highly powered datasets. **f,g)** Analogs of (c,d) but simulated with power matched that of the cohort studies in Kamphaeng Phet. Center of each panel compares inferred temporal force of infection from seroincidence data (red) and seroprevalence data (yellow) to ground truth (black). Right of the panels are scatter plots between inferred infection risk and true infection risk by age.

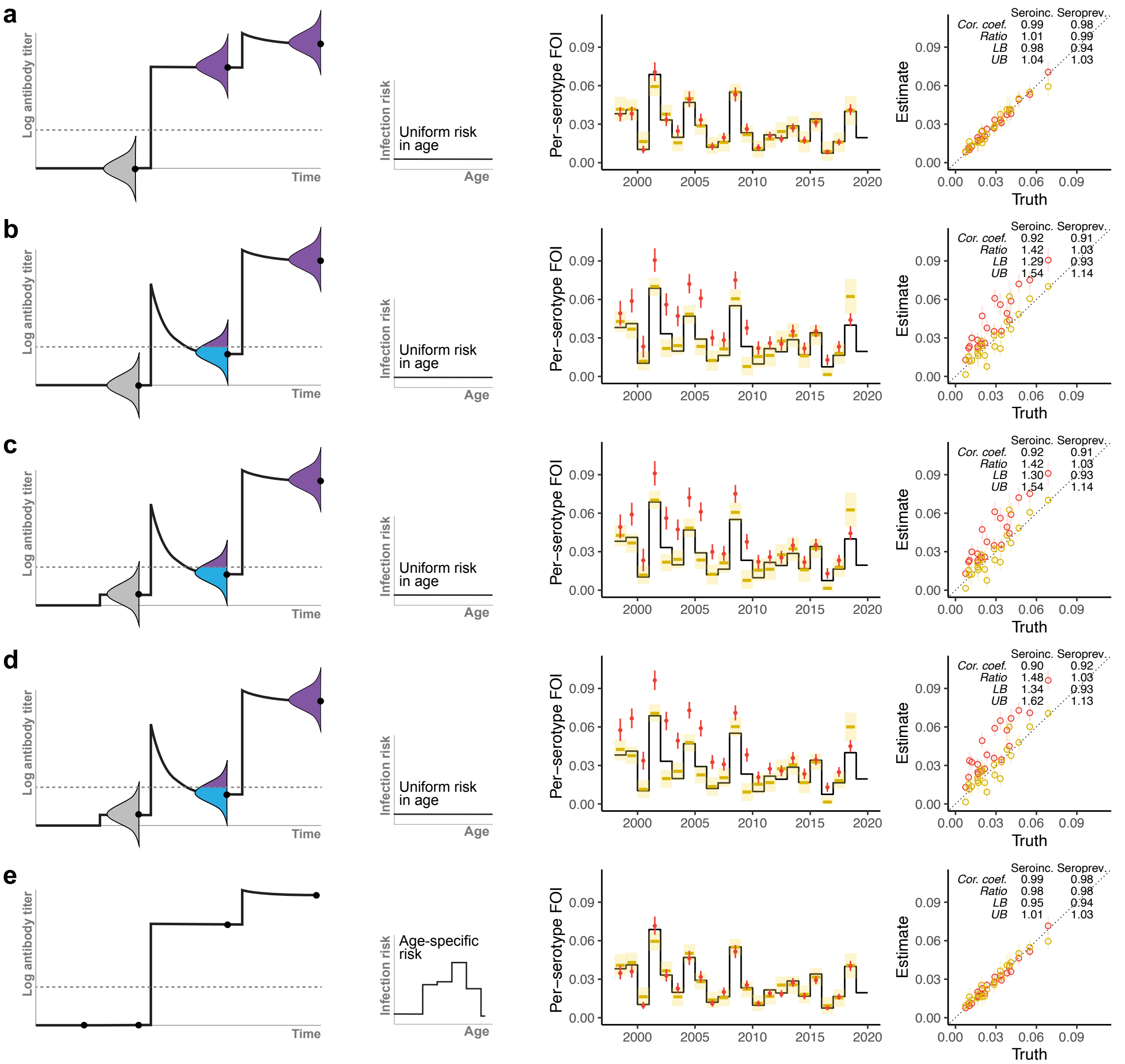

Figure S7. Efficiency in correcting for model assumption violations to recover true temporal infection risk when using a high seropositivity threshold (GMT>=20). Left of each panel are schematics of assay variability, antibody kinetics, seropositivity thresholds, and age-specific infection risk used to simulate the data: **a)** noisy assay, durable monotypic titers, without cross-reactive titers, uniform risk in age **b)** noisy assay, waning monotypic titers, without cross-reactive titers, uniform risk in age **c)** noisy assay, waning monotypic titers, with cross-reactive titers, uniform risk in age **d)** noisy assay, waning monotypic titers, with cross-reactive titers, non-uniform risk in age, **e)** assay without noise, durable monotypic titers, without cross-reactive titers, non-uniform risk in age. All of which were simulated as highly powered datasets. Center of each panel compares inferred temporal force of infection from seroincidence data (red) and seroprevalence data (yellow) to ground truth (black). Right of the panels are scatter plots between inferred infection risk and true infection risk by age.

Extended models to reconcile infection risk estimates

Table S3. Priors of parameters in the joint serology model.

Parameter	Description	Prior	Prior justification
$\tau(t)$	Annual per-serotype force of infection faced by individuals in the reference age class	Exponential(2)	Weak prior
$\kappa(a)$	Force of infection faced by a specific age class relative to individuals aged 0-5yrs (reference class)	Lognormal(0, 0.1)	Strong prior to favor infection risk to be uniform across age unless data suggests otherwise
$\Omega_{short,ref}$	Short-term titer rise captured by post-interval bleeds of KPS1 for a 1st infection that occurred within the interval	Gamma(6.399141, 1.187225)	Priors taken/solved from means and variances reported in Salje, 2018 (1)
$\Omega_{short,rel}(z)$	Short-term titer rise captured by post-interval bleeds of study z for a 1st infection that occurred within the interval relative to KPS1	Lognormal(0, 0.1)	Strong prior to favor consistent short-term titer rise across studies unless data suggests otherwise
Ω_{long}	Long-term titer rise after 1st infection of individuals	Gamma(0.9561622, 0.7189189)*	Priors taken/solved from means and variances reported in Salje, 2018 (1)
$\Omega_{0,rel}$	Cross-reactive titer in DENV-naive individuals relative to Ω_{long}	Beta(1,9)	Weakly informative prior
σ	Standard deviation of titer measurements in sera of DENV-exposed individuals	Normal(0.49, 0.1)*	Priors taken/solved from means and variances reported in Salje, 2018 (1)

Table S4. Priors of parameters in the extended case-based model.

Parameter	Description	Prior	Prior justification
$\tau(t)$	Annual force of infection faced by individuals in the reference age class	Exponential(2)	Weak prior
$\kappa(a)$	Force of infection faced by a specific age class relative to individuals aged 0-2yrs (reference class)	Lognormal(0, 0.1)	Strong prior to favor infection risk to be uniform across age unless data suggests otherwise
$p_{severe}(1)$	Probability that 1st infections of individuals resulted in severe infections relative to 2nd infections	Beta(1, 9)	Weakly informative prior to encode knowledge of 1st dengue infections being relatively mild compared to 2nd infections
$p_{severe}(i)$	Probability that i-th infections (3rd or 4th) of individuals resulted in severe infections relative to 2nd infections	Beta(1, 19)	Informative prior to reflect the rare occurrence of severe dengue in 3rd and 4th infections
$\phi(a)$	Probability that a severe case of age a sought care at KPPH relative to cases of age 0-2yrs (reference class)	Lognormal(0, 0.1)	Informative prior to favor reporting to be constant in age unless data suggests otherwise
$\phi(t)$	Probability that a severe case of age 0-2yrs sought care at KPPH at year t	Beta(2,2)	Weak prior

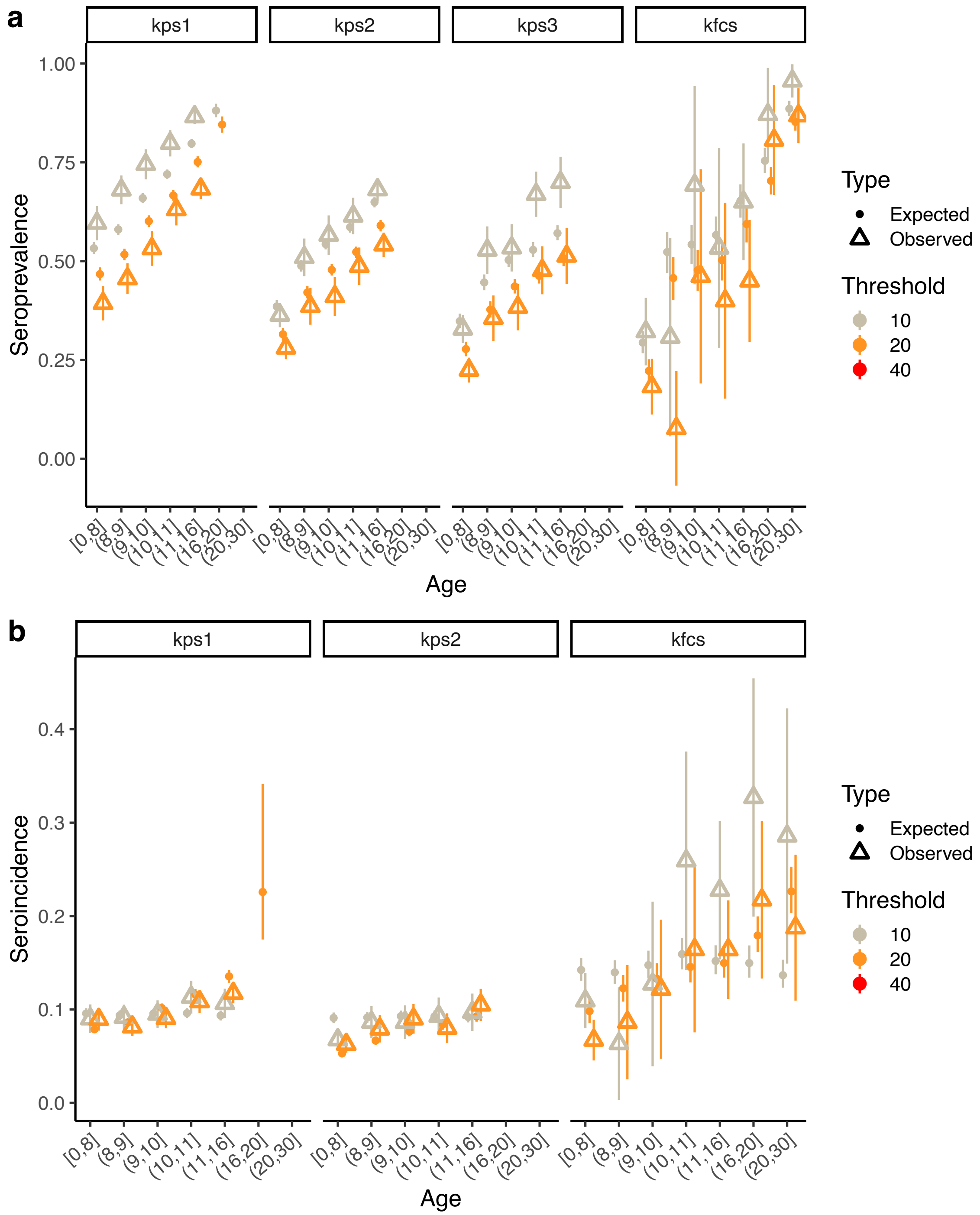

Figure S8. Observed serology data vs expectations from the joint serology model fit. a) Seropositive proportions by age group and study using seropositivity thresholds of GMT>=10 (gray) and 20 (orange) compared against their respective expectations from the model fits (lines). **b)** Seroincidence by age group and study using seropositivity threshold of 10.

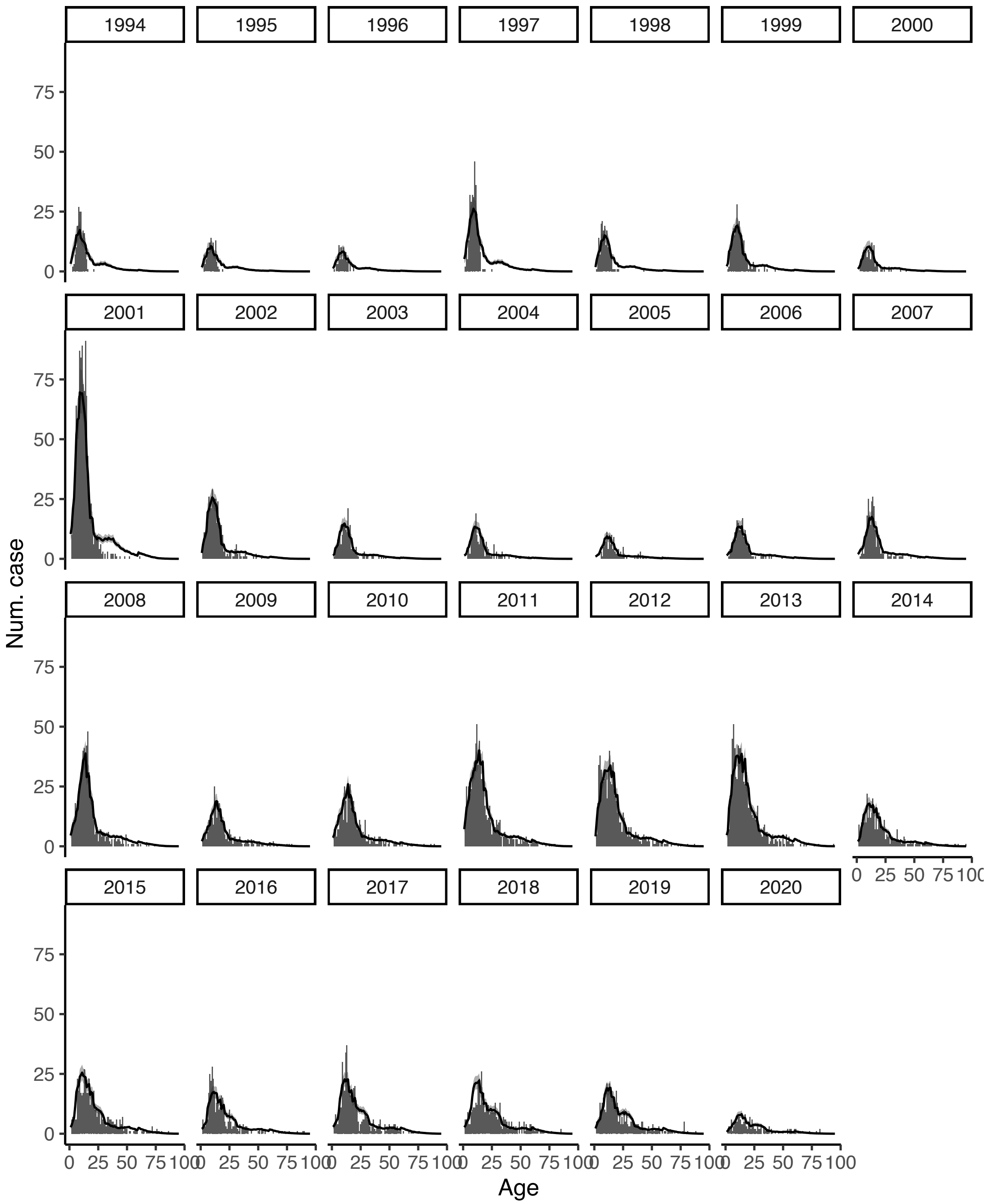

Figure S9. Observed case data vs expectations from the extended case-based model fit. Number of reported dengue cases at KPPH by age and year (bars) compared against its expectations from the model fits (lines).

Table S5. Posterior medians (and 95% credible intervals) of infection parameters in the joint serology model and extended case-based model.

Parameter and description	Year / Age group	Posterior median of joint serology model (95% credible interval)	Posterior median of case-based model (95% credible interval)
$\tau(t)$	Up to 1984	0.139 (0.006, 0.565)	0.040 (0.034, 0.048)
	1985	0.042 (0.002, 0.171)	0.180 (0.061, 0.303)
	1986	0.013 (0.000, 0.061)	0.149 (0.033, 0.272)
Annual per-serotype force of infection faced by individuals in the reference age class	1987	0.075 (0.023, 0.125)	0.049 (0.003, 0.149)
	1988	0.032 (0.002, 0.083)	0.021 (0.001, 0.087)
	1989	0.075 (0.032, 0.111)	0.030 (0.001, 0.110)
	1990	0.023 (0.001, 0.059)	0.030 (0.001, 0.101)
	1991	0.022 (0.002, 0.053)	0.024 (0.001, 0.093)
	1992	0.026 (0.002, 0.059)	0.012 (0.000, 0.059)
	1993	0.045 (0.018, 0.071)	0.016 (0.001, 0.075)
	1994	0.006 (0.000, 0.023)	0.038 (0.003, 0.091)
	1995	0.010 (0.001, 0.033)	0.022 (0.002, 0.053)
	1996	0.034 (0.008, 0.059)	0.017 (0.003, 0.035)
	1997	0.015 (0.001, 0.038)	0.050 (0.011, 0.097)
	1998	0.032 (0.023, 0.041)	0.010 (0.001, 0.031)
	1999	0.027 (0.020, 0.035)	0.014 (0.002, 0.039)
	2000	0.001 (0.000, 0.003)	0.006 (0.002, 0.012)
	2001	0.031 (0.024, 0.038)	0.044 (0.017, 0.073)
	2002	0.001 (0.000, 0.004)	0.007 (0.001, 0.027)
	2003	0.035 (0.023, 0.046)	0.004 (0.001, 0.016)
	2004	0.002 (0.000, 0.006)	0.007 (0.001, 0.023)
	2005	0.006 (0.002, 0.011)	0.005 (0.001, 0.016)
	2006	0.015 (0.011, 0.020)	0.007 (0.001, 0.019)
	2007	0.006 (0.003, 0.010)	0.009 (0.001, 0.024)
	2008	0.012 (0.000, 0.037)	0.014 (0.003, 0.041)
	2009	0.009 (0.000, 0.032)	0.007 (0.001, 0.021)
	2010	0.045 (0.009, 0.078)	0.037 (0.023, 0.056)
	2011	0.013 (0.001, 0.049)	0.061 (0.040, 0.091)
	2012	0.015 (0.001, 0.059)	0.007 (0.002, 0.017)
	2013	0.023 (0.001, 0.075)	0.008 (0.003, 0.020)
	2014	0.017 (0.001, 0.061)	0.005 (0.001, 0.014)
	2015	0.003 (0.000, 0.012)	0.007 (0.002, 0.019)
	2016	0.002 (0.000, 0.008)	0.004 (0.001, 0.014)
	2017	0.001 (0.000, 0.005)	0.005 (0.002, 0.018)
	2018	0.044 (0.033, 0.054)	0.028 (0.011, 0.055)
	2019	0.010 (0.000, 0.040)	0.024 (0.010, 0.047)
	2020	n/a	0.010 (0.004, 0.020)
$\kappa(a)$	0-2 yrs	Ref.	Ref.
	3-5 yrs	Ref.	1.075 (0.938, 1.230)
	6-8 yrs	1.189 (1.010, 1.390)	1.271 (1.101, 1.466)
Force of infection faced by a specific age class relative to the reference class	9-11 yrs	1.291 (1.124, 1.477)	1.284 (1.115, 1.463)
	12-14 yrs	1.093 (0.912, 1.303)	1.333 (1.151, 1.536)
	15-17 yrs	1.205 (1.003, 1.448)	1.174 (1.015, 1.345)
	18-20 yrs	1.000 (1.000, 1.000)	0.980 (0.845, 1.135)
	21-23 yrs	n/a	0.848 (0.723, 0.999)
	24-26 yrs	n/a	0.819 (0.692, 0.960)
	27-29 yrs	n/a	0.834 (0.707, 0.982)

	30-39 yrs<br>40-49 yrs<br>50-59 yrs<br>60-62 yrs<br>63+ yrs	n/a<br>n/a<br>n/a<br>n/a<br>n/a	0.838 (0.714, 0.970)<br>0.818 (0.708, 0.952)<br>0.888 (0.767, 1.030)<br>1.137 (0.961, 1.335)<br>1.000 (1.000, 1.000)

Table S6. Posterior medians (and 95% credible intervals) of parameters in the joint serology model linking infection risk to serological data.

Parameter	Description	Posterior median (95% credible interval)
$\Omega_{short,ref}$	Short-term titer rise captured by post-interval bleeds of KPS1 for a 1st infection that occurred within the interval	7.826 (4.786, 13.058)
$\Omega_{short,rel}(z)$	Short-term titer rise captured by post-interval bleeds of study z for a 1st infection that occurred within the interval relative to KPS1	KPS2: 1.000 (0.817, 1.211)<br>KPS3: 1.000 (0.820, 1.213)<br>KFCS: 0.999 (0.822, 1.227)
Ω_{long}	Long-term titer rise after 1st infection of individuals	2.735 (2.510, 2.935)
$\Omega_{0,rel}$	Cross-reactive titers in DENV-naive individuals relative to long-term titer rise after 1st infection of individuals	0.093 (0.017, 0.192)
σ	Standard deviation of titer measurements in sera of DENV-exposed individuals	0.512 (0.359, 0.646)

Table S7. Posterior medians (and 95% credible intervals) of parameters in the case-based model linking infection risk to case data.

Parameter	Description	Posterior median (95% credible interval)	
$p_{severe}(i)$	Probability that i-th infections of individuals resulted in severe infections relative to 2nd infections	i=1	0.066 (0.030, 0.104)
		i=2	Ref.
		i=3	0.010 (0.000, 0.053)
		i=4	0.331 (0.248, 0.426)
$\phi(a)$	Probability that a severe case of age a sought care at KPPH relative to cases of age 0-2yrs (reference class)	0-2	Ref.
		3-5	1.032 (0.887, 1.212)
		6-8	1.111 (0.948, 1.311)
		9-11	1.234 (1.059, 1.424)
		12-14	1.139 (0.981, 1.319)
		15-17	1.044 (0.900, 1.209)
		18-20	0.912 (0.784, 1.057)
		21-23	0.762 (0.653, 0.881)
		24-26	0.785 (0.673, 0.915)
		27-29	0.830 (0.708, 0.978)
		30-39	0.873 (0.745, 1.023)
		40-49	0.946 (0.809, 1.116)
		50-59	1.025 (0.873, 1.197)
		60+	1.383 (1.175, 1.633)
$\phi(t)$	Probability that a severe case at age 0-2yrs sought care at KPPH at year t	1994-1995	0.022 (0.008, 0.286)
		1996-1997	0.022 (0.011, 0.098)
		1998-1999	0.058 (0.020, 0.474)
		2000-2001	0.067 (0.039, 0.170)
		2002-2003	0.148 (0.037, 0.690)
		2004-2005	0.080 (0.024, 0.613)
		2006-2007	0.084 (0.032, 0.593)
		2008-2009	0.119 (0.041, 0.617)
		2010-2011	0.030 (0.020, 0.048)
		2012-2013	0.245 (0.105, 0.717)
		2014-2015	0.199 (0.071, 0.694)
		2016-2017	0.229 (0.067, 0.737)
		2018-2020	0.042 (0.022, 0.102)

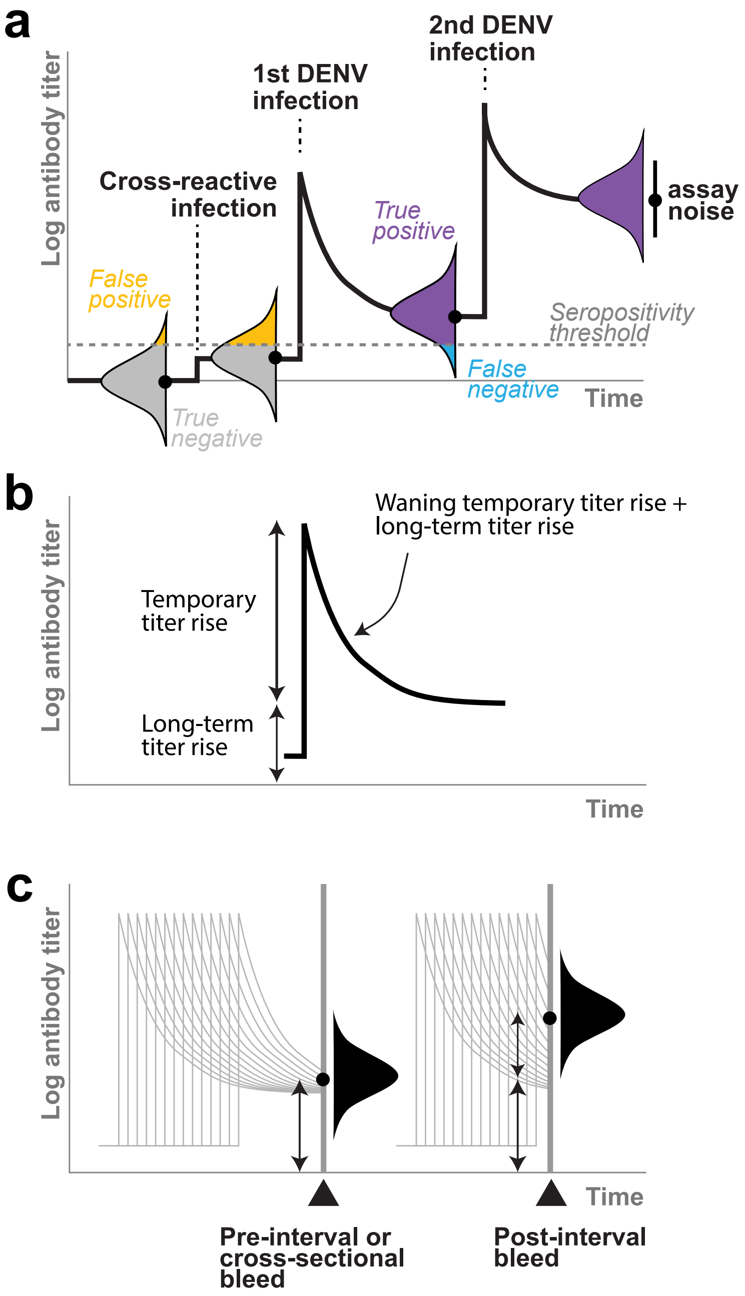

Figure S10. Relationship between titer parameters in the joint serology model and individual-level anti-DENV antibody kinetics described Figure 2 and in the Supplementary Mathematical Analysis. **a)** Illustration of anti-DENV antibody kinetics as an individual acquires a cross-reactive (CXR) virus infection or vaccination (i.e., not DENV), one DENV infection, and >1 DENV infections. Measured titers distribute around the true underlying titers with variability depending on the assay characteristics. **b)** Titer components in the antibody kinetics upon first DENV infection of an individual. **c)** Titer parameters in the joint serology model encodes the average long-term titer rises captured in pre-interval or cross-sectional blood samples (Ω_{long}) across individuals, and the average titer rises captured in post-interval blood samples as a result of infections that occurred during the interval ($\Omega_{long} + \Omega_{short}$).

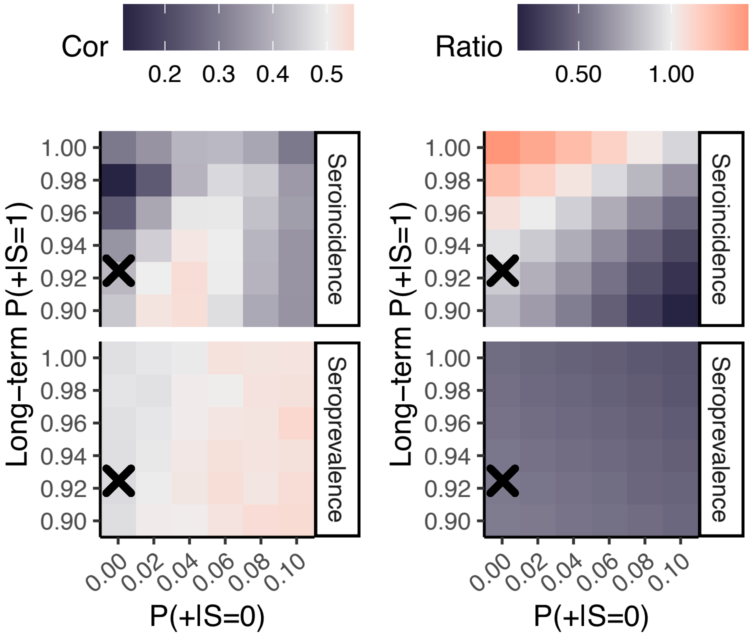

Figure S11. Variations in FOI congruence across presumed test positive probabilities. Effects of presumed test positivity probabilities on the correlation and ratio between temporal FOIs inferred from the extended case-based model and temporal FOIs inferred from a single data source (either seroincidence or seroprevalence at seropositivity threshold of 20) imposed with age-specific risk inferred from the extended case-based model. Test positive probabilities estimated from the joint serology model are annotated as crosses for comparison.

SI References

1. H Salje, et al., Reconstruction of antibody dynamics and infection histories to evaluate dengue risk. *Nature* 557, 719–723 (2018).

Supplementary mathematical analysis: biases in force of infection inferred from seroincidence data

Notations used throughout this document are defined as follows.

$$
\begin{aligned}
P(\ominus_t) &= \text{Probability of testing negative at time } t \\
P(\oplus_t) &= \text{Probability of testing positive at time } t \\
P(\ominus_t^T) &= \text{Probability of being true negative at time } t \\
P(\oplus_t^T) &= \text{Probability of being true positive at time } t \\
P(\ominus_t^F) &= \text{Probability of being false negative at time } t \\
P(\oplus_t^F) &= \text{Probability of being false positive at time } t \\
P(S_t = i) &= \text{Probability of having acquired } i \text{ infections at time } t \\
p_a &= \text{Probability of escaping a particular serotype up till age } a \\
\bar{\lambda} &= \text{Average historical per-serotype force of infection} \\
\lambda_t &= \text{Per-serotype force of infection during time interval } (t, t+\Delta t] \\
\omega &= \text{Long-term persistent titer rise acquired upon primary dengue infection} \\
\gamma &= \text{Temporary titer rise upon primary dengue infection} \\
\delta &= \text{Rate of exponential decay in temporary titers} \\
\sigma &= \text{Standard deviation of assay measurements from true underlying titer of a DENV-exposed individual} \\
\sigma_0 &= \text{Standard deviation of assay measurements from true underlying titer of a DENV-naive individual} \\
\nu &= \text{Seropositivity titer cut-off} \\
\Phi(x) &= \text{Cumulative density of a standard normal distribution at value } x
\end{aligned}
$$

Longitudinal serology is typically viewed as the gold standard for measuring levels of transmission within a time period as it directly measures serological changes of individuals. We can express the **true probability of first infection incidence** during a time interval $(t, t + \Delta t]$ as

$$P(S_{t+\Delta t} \geq 1 | S_t = 0) = P(S_{t+\Delta t} = 1 | S_t = 0) + P(S_{t+\Delta t} > 1 | S_t = 0) \tag{1}$$

Probability of observing seroconversion in an individual, $P(\oplus_{(t,t+\Delta t]} | \ominus_t)$, can be expanded to a weighted average between probabilities of seroconverting given being truely negative at the first time point, t, and being falsely negative.

$$P(\oplus_{(t,t+\Delta t]} | \ominus_t) = \frac{P(\ominus_t^T)}{P(\ominus_t)} [P(\oplus_{t+\Delta t}^T | \ominus_t^T) + P(\oplus_{t+\Delta t}^F | \ominus_t^T)] + \frac{P(\ominus_t^F)}{P(\ominus_t)} [P(\oplus_{t+\Delta t}^T | \ominus_t^F) + P(\oplus_{t+\Delta t}^F | \ominus_t^F)] \tag{2}$$

Biases in seroconversion probabilities when truly negative at t from the true probability of first infection incidence is then

$$
\begin{aligned}
&[P(\oplus^T_{(t,t+\Delta t]}|\ominus^T_t) + P(\oplus^F_{(t,t+\Delta t]}|\ominus^T_t)] - P(S_{(t,t+\Delta t]} = 1|S_t = 0) - P(S_{(t,t+\Delta t]} > 1|S_t = 0) \\
&= P(S_{(t,t+\Delta t]} = 1|S_t = 0)\ P(\oplus^T_{(t,t+\Delta t]}|S_{(t,t+\Delta t]} = 1) + P(S_{(t,t+\Delta t]} > 1|S_t = 0)\ P(\oplus^T_{(t,t+\Delta t]}|S_{(t,t+\Delta t]} > 1) \\
&+ P(S_{(t,t+\Delta t]} = 0|S_t = 0)\ P(\oplus^F_{(t,t+\Delta t]}|S_{(t,t+\Delta t]} = 0) - P(S_{(t,t+\Delta t]} = 1|S_t = 0) - P(S_{(t,t+\Delta t]} > 1|S_t = 0) \\
&= P(S_{(t,t+\Delta t]} = 0|S_t = 0)\ P(\oplus^F_{(t,t+\Delta t]}|S_{(t,t+\Delta t]} = 0) \\
&+ P(S_{(t,t+\Delta t]} = 1|S_t = 0)[P(\oplus^T_{(t,t+\Delta t]}|S_{(t,t+\Delta t]} = 1) - 1] \\
&+ P(S_{(t,t+\Delta t]} > 1|S_t = 0)[P(\oplus^T_{(t,t+\Delta t]}|S_{(t,t+\Delta t]} > 1) - 1]
\end{aligned} \tag{3}
$$

We can see that the bias is a weighted average between biases when no infection occurred during the interval ($S_{(t,t+\Delta t]} = 0, S_t = 0$), when one infection occurred during the interval ($S_{(t,t+\Delta t]} = 1|S_t = 0$), and when more than one infection occurred during the interval ($S_{(t,t+\Delta t]} > 1|S_t = 0$). Assuming long-lived protection against infecting serotypes and no cross-protection between the serotypes, we can write the probabilities of these scenarios (i.e., the weights) for an individual of age a at time $(t, t + \Delta t]$ as

$$
P(S_{(t,t+\Delta t]} = i) = \binom{4}{i}\ (e^{-\bar{\lambda} a})^{4-i}\ (1 - e^{-\bar{\lambda} a})^i \tag{4}
$$

Previous studies on the kinetics of anti-DENV antibodies have shown that the rise in antibody levels after having acquired one infection, i.e., monotypic titers, consists of a portion which diminishes within a year and a portion which persists for longer (cite: Leah, Henrik). Following Salje et al, the kinetics of H_t, the true titer at time t, can be characterized as

$$
H_t = \omega + \gamma e^{-(t - \star t)\delta} \tag{5}
$$

where ω is the long-term persistent titer acquired upon infection, γ is the temporary titer rise, δ is the rate of exponential decay in temporary titer rise, and $\star t$ is time at which the infection occurred. For an assay with measurement noise which follows a normal distribution $N(0, \sigma^2)$, using seropositivity cut-off ν, the probability of testing positive after being infected once is

$$
P(\oplus^T_t|S_t = 1) = 1 - \Phi(\frac{\nu - H_t}{\sigma}) \tag{6}
$$

In DENV-naive individuals, although anti-DENV antibodies are absent, cross-reactive titers against other *Flaviviruses* in circulation could plausibly result in non-zero titers against DENV, ω_0. We can, in a similar manner, express the probability of testing positive when DENV-naive as

$$
P(\oplus^F_t|S_t = 0) = 1 - \Phi(\frac{\nu - \omega_0}{\sigma}) \tag{7}
$$

Let probability of escaping a particular serotype during time interval $e^{-\lambda_t \Delta t} = p$, we can express (3) as

$$
\begin{aligned}
&p^4\ [1 - \Phi(\frac{\nu - \omega_0}{\sigma})] + \binom{4}{1}\ p^3\ (1-p)\ [(1 - \Phi(\frac{\nu - H_t}{\sigma})) - 1] + [1 - p^4 - \binom{4}{1}\ p^3\ (1-p)]\ [1 - 1] \\
&= p^4\ [1 - \Phi(\frac{\nu - \omega_0}{\sigma})] - 4\ p^3\ (1-p)\ \Phi(\frac{\nu - H_t}{\sigma}))
\end{aligned} \tag{8}
$$

For the first case in Equation (3) where no infection occurred, the bias increases when the seropostivity threshold is lowered. In the second case where only one infection occurred, the bias becomes less negative when time since infection is small or when the seropostivity threshold is lowered. Since time since infection

in this case is bounded by the interval length, shorter bleeding intervals are more likely to be less negative. Titers in individuals who have acquired multiple DENV infections, i.e., multitypic titers, are more robust and can be assumed to test negative at negligible levels. It follows that minimal bias would arise from the last case where more than one infection occurred during the interval.

Biases in seroconversion probabilities when falsely negative at t, under these described processes, can be expressed as

$$\begin{aligned}
&P(\oplus^T_{(t,t+\Delta t]}|\ominus^F_t) + P(\oplus^F_{(t,t+\Delta t]}|\ominus^F_t) - P(S_{(t,t+\Delta t]} = 1|S_t = 0) - P(S_{(t,t+\Delta t]} > 1|S_t = 0) \\
&= P(\oplus^T_{(t,t+\Delta t]}|\ominus_t, S_t = 1) + 0 - P(S_{(t,t+\Delta t]} = 1|S_t = 0) - P(S_{(t,t+\Delta t]} > 1|S_t = 0) \\
&= P(\oplus_{(t,t+\Delta t]}, S_{(t,t+\Delta t]} = 1|\ominus_t, S_t = 1) + P(\oplus^T_{(t,t+\Delta t]}, S_{(t,t+\Delta t]} > 1|\ominus^F_t, S_t = 1) \\
&- P(S_{(t,t+\Delta t]} = 1|S_t = 0) - P(S_{(t,t+\Delta t]} > 1|S_t = 0)
\end{aligned} \tag{9}$$

The first case where $S_{(t,t+\Delta t]} = S_t = 1$ means that no infection occurred during the interval. $P(\oplus_{(t,t+\Delta t]})$ in this case has a lowerbound of $1 - \Phi(\frac{\nu-\omega}{\sigma})$. In the second case where infection(s) occurred during the interval, $S_t = 1$ and $S_{(t,t+\Delta t]} > 1$, $P(\oplus_{(t,t+\Delta t]}) \approx 1$. Hence, $P(\oplus^T_{(t,t+\Delta t]}|\ominus^F_t)$ is a weighted average between two quantities, $1 - \Phi(\frac{\nu-\omega}{\sigma})$ and 1. Longer time between blood draws, Δt, and higher FOI, λ_t, during the interval tends the quantity towards 1. Again, for probability of escaping a particular serotype during the interval $p = e^{-\lambda_t \Delta t}$, we can express (9) as

$$\begin{aligned}
&p^3 * (1 - \Phi(\frac{\nu - H_t}{\sigma})) + (1 - p^3) - (1 - p^4) \\
&= p^4 - \Phi(\frac{\nu - H_t}{\sigma})p^3
\end{aligned} \tag{10}$$

Relative contributions of the biases can be expressed as

$$\begin{aligned}
\frac{P(\ominus^F_t)}{P(\ominus^T_t)} &= \frac{P(S_t = 1)\ P(\ominus^F_t|S_t = 1)}{P(S_t = 0)\ P(\ominus^T_t|S_t = 0)} \\
&= \frac{\binom{4}{1}\ (e^{-\bar{\lambda}a})^3\ (1 - e^{-\bar{\lambda}a})}{(e^{-\bar{\lambda}a})^4}\ \frac{P(\ominus^F_t|S_t = 1)}{P(\ominus^T_t|S_t = 0)} \\
&= 4\ \frac{(1 - e^{-\bar{\lambda}a})}{(e^{-\bar{\lambda}a})}\ \frac{P(\ominus^F_t|S_t = 1)}{P(\ominus^T_t|S_t = 0)}
\end{aligned} \tag{11}$$

The quantity is a product of the constant 4 and two variable components. The first variable component is zero when a is zero or the average per-serotype force of infection $\bar{\lambda}$ is zero and grows as the product $\bar{\lambda}a$ increases. The second variable component depends on the recency of the dengue infection with bounds

$$\frac{\Phi(\frac{\nu-\omega-\gamma}{\sigma})}{\Phi(\frac{\nu-\omega_0}{\sigma})} \le \frac{P(\ominus^F_t|S_t = 1)}{P(\ominus^T_t|S_t = 0)} \le \frac{\Phi(\frac{\nu-\omega}{\sigma})}{\Phi(\frac{\nu-\omega_0}{\sigma})} \le 1 \tag{12}$$

Assuming cross-reactive titer ω_0 is at most ω, the quantity is bounded at one. Intuitively, lowering the positivity cut-off ν would decrease contribution of $\ominus^F_t$ to $\ominus_t$. However, the contribution can only be minimized down to $1/(1 + \Phi(\frac{\nu-\omega_0}{\sigma})/\Phi(\frac{\nu-\omega-\gamma}{\sigma}))$ and the efficiency of such reduction declines with age and historical FOI $\bar{\lambda}$.

Figure S12 illustrates ranges of the biases and their relative contributions using mean parameter estimates of $\omega = 1.33$ and $\sigma = 0.49$ from Salje, 2018 under various interval lengths and seropositivity cut-offs. The amount of cross-reactive titers relative to DENV-specific titers was artibrarily set to 20% ($\omega_0 = 0.2\omega$). Per-serotype FOI (historical and during the interval) is set to 0.03. Under these parameters, we can see that the magnitude

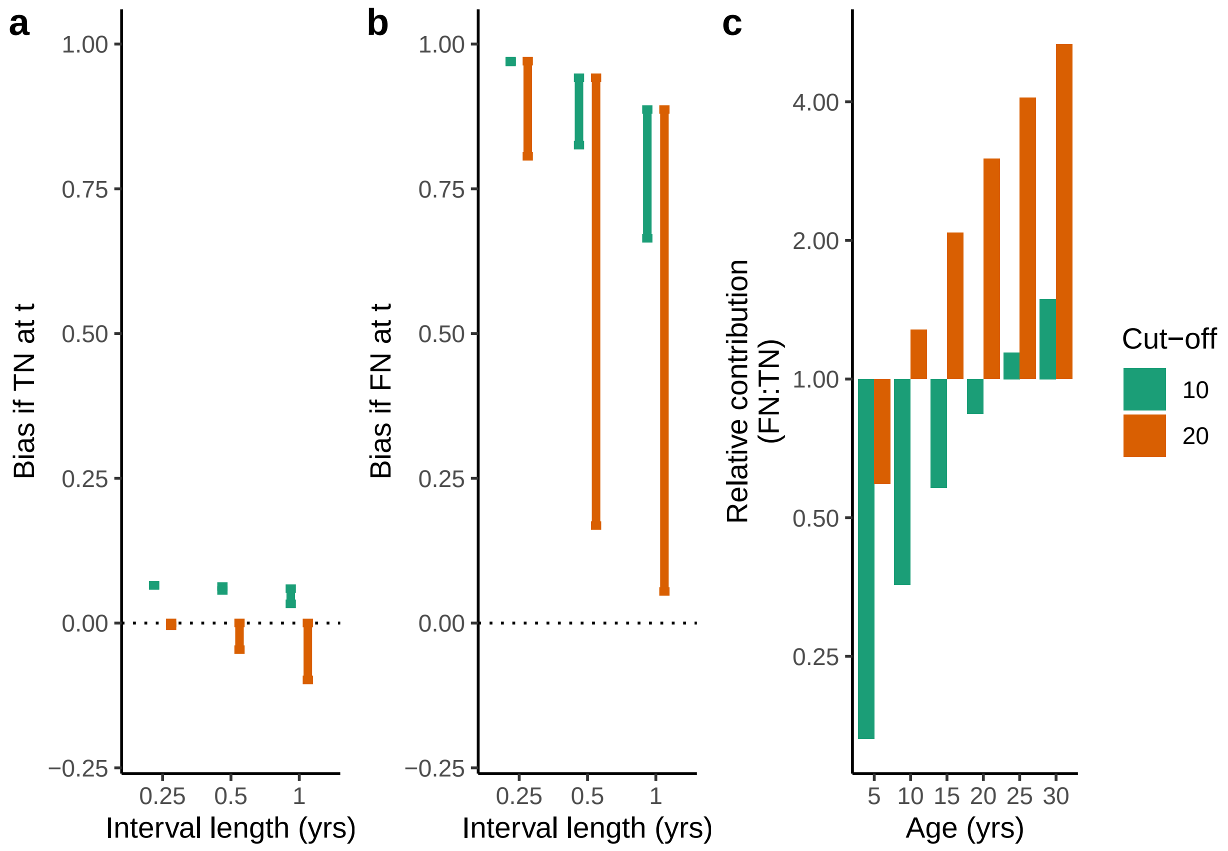

Figure S12: Biases in seroconversion probabilities illustrated using antibody kinetics estimates from Salje, 2018. Biases in the case where the individual was a) true negative (TN) at pre-interval bleed versus b) false negative (FN) at pre-interval bleed. c) Upper bound of relative contribution between the FN and TN case to the seroconversion probability as a function of pre-interval age for seropositivity cut-offs 10 and 20.

of positive bias from false negative scenario at t is far higher biases from the true negative scenario. Increasing the cut-off reduces the lower bound of such inflation (leading to greater uncertainty in the amount of bias introduced) and increases the contribution of the false negative scenario.

Effects of seasonality on test positivity. The derivations, so far, assumes that FOI is constant throughout the year. However, it is conceivable that timings of the bleeds in relation to phases in the dengue seasons would lead to non-uniform distribution of the infections, and hence, may influence the expected biases.

Let the per-serotype FOI at age a of an individual $\lambda(a) = \bar{\lambda} + \delta_\lambda cos(2\pi(a + \delta_t))$ where δ_λ is the magnitude of fluctuation and δ_t governs the phase of the fluctuation in relation to age. The per-serotype infection risk faced up till age A is then

$$\Lambda(A) = \int_0^A \bar{\lambda} + \delta_\lambda cos(2\pi(a + \delta_t))\, da = \bar{\lambda}A + \frac{\delta_\lambda}{2\pi}[sin(2\pi(A + \delta_t)) - sin(2\pi\delta_t)] \tag{13}$$

We can write the probability that the individual was infected by a particular serotype at age A as $e^{-\Lambda(A)}\lambda(A)$. Thus, the expected underlying titer of the individual at age A_1 given the individual was monotypically infected at that point is

$$\begin{aligned} \mathbb{E}[H_{A_1}|S_{A_1} = 1] &= \frac{3\ e^{-\Lambda(A_1)}\ \int_0^{A_1}(\omega + \gamma e^{-\delta(A_1 - A)})\ e^{-\Lambda(A)}\lambda(A)\, dA}{3\ e^{-\Lambda(A_1)}\ (1 - e^{-\Lambda(A_1)})} \\ &= \frac{\int_0^{A_1}(\omega + \gamma e^{-\delta(A_1 - A)})\ e^{-\Lambda(A)}\lambda(A)\, dA}{1 - e^{-\Lambda(A_1)}} \end{aligned} \tag{14}$$

The growing contribution of $\bar{\lambda}A$ in $\Lambda(A)$ as age increases shrinks the impact of timing of the pre-interval blood draw in relation to seasonality. Figure S13a-c illustrates this shrinkage under parameter estimates from Salje, 2018. On the other hand, for infections that occurred within the intervals, seasonality and the interval lengths dictates the distribution of time since infection at the second bleed (Figure S13d). These timing variations affects the amount of titers waned, and consequentially, the probability of falsely testing negative at post-interval bleed. Figure S13e illustrates this nuance under parameter estimates from Salje, 2018.

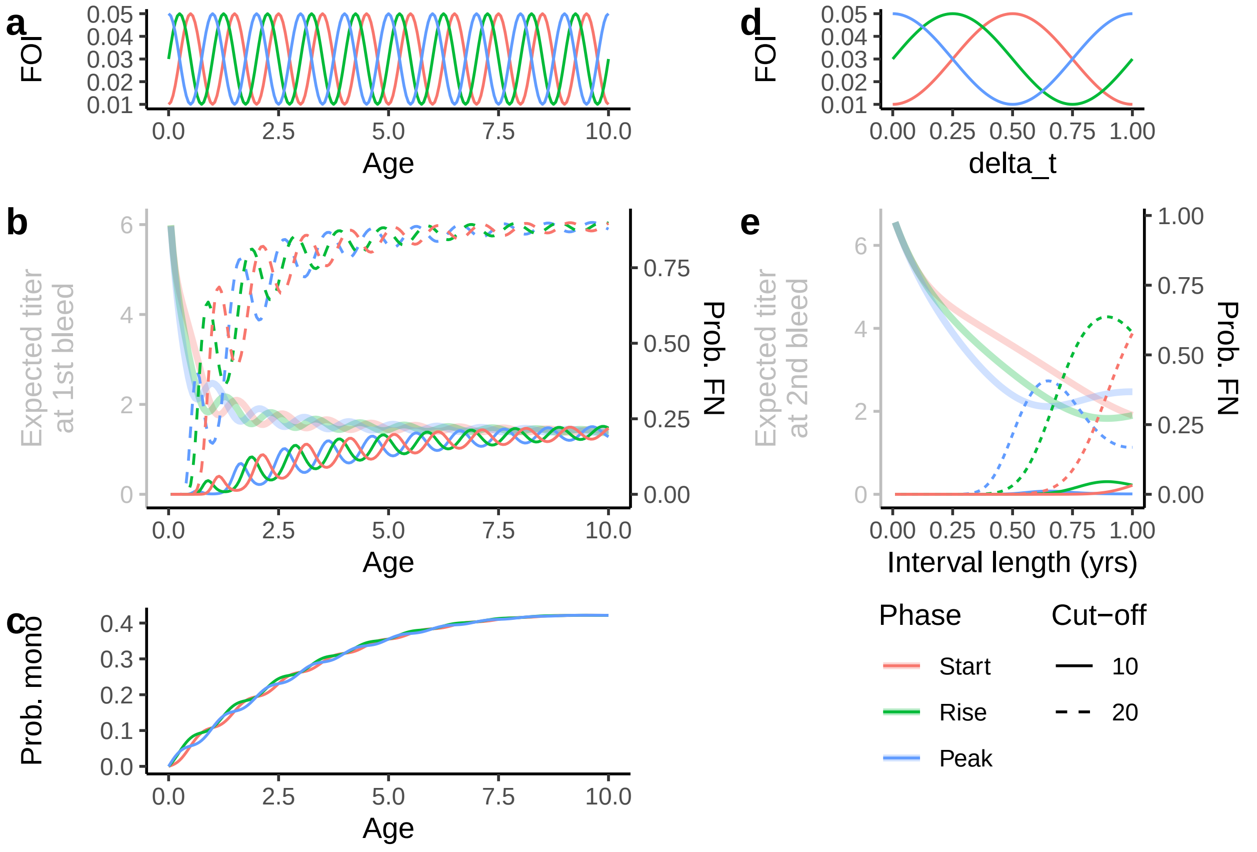

Figure S13: Effects of timings of bleeds in relation to seasonality illustrated using antibody kinetics estimates from Salje, 2018. a) Per-serotype seasonal force of infection (FOI) in age. b) Expected pre-interval titer of a monotypically infected individual (tinted) and the corresponding probability of falsely testing negative given the titers at positivity threshold of 10 (solid) and 20 (dashed). c) Probability that the individual was monotypically infected. d) Relationship between per-serotype seasonal force of infection (FOI) and years since pre-interval bleed. e) Expected post-interval titer of individuals that acquired their first infection during the interval (tinted) and the corresponding probability of falsely testing negative given the titers at positivity threshold of 10 (solid) and 20 (dashed).
